# Temporal trend and epidemiological profile of accidents involving venomous animals in Brazil, 2007-2019

**DOI:** 10.1590/S2237-96222022000300009

**Published:** 2022-11-04

**Authors:** Tiago Cruz de Souza, Beatrice Emeli Silva Farias, Paulo Sérgio Bernarde, Francisco Chiaravalotti, Djair Durand Ramalho Frade, Andreia Fernandes Brilhante, Leonardo Augusto Kohara Melchior

**Affiliations:** 1 Universidade Federal do Acre, Programa de Pós-Graduação em Ciências da Saúde na Amazônia Ocidental, Rio Branco, AC, Brazil; 2 Universidade Federal do Acre, Centro de Ciências da Saúde e do Desporto, Rio Branco, AC, Brazil; 3 Universidade Federal do Acre, Laboratório de Herpetologia, Cruzeiro do Sul, AC, Brazil; 4 Universidade de São Paulo, Faculdade de Saúde Pública, São Paulo, SP, Brazil; 5 Universidade Federal do Acre, Centro de Ciências Exatas e Tecnológicas, Rio Branco, AC, Brazil

**Keywords:** Snake Bites, Spatio-Temporal Analysis, Scorpions, Spider Bites, Health Information Systems

## Abstract

**Objective:**

to analyze the temporal trend of accidents involving venomous animals in Brazil from 2007 to 2019.

**Methods:**

this was a cross-sectional study carried out with data from the Notifiable Health Conditions Information System (SINAN). Prais-Winsten linear regression was used for the temporal analysis. We calculated incidence rates according to sex and age group, relative risk and case fatality ratio.

**Results:**

during the study period there were 2,102,657 cases of accidents involving venomous animals. With the exception of snakebite, the remaining accidents showed a rising temporal trend in most regions of the country. Scorpion stings, snake bites and spider bites were responsible for 86% of accidents, mainly affecting male people of working age. Accidents involving snakes (0.4%) and bees (0.3%) had the highest case fatality ratios. Children were the main victims of accidents involving bees, caterpillars and "others".

**Conclusion:**

accidents involving venomous animals showed a rising temporal trend for most conditions, as well as different epidemiological profiles.

**Study contributions**

**Main results**

With the exception of snake bites, the remaining accidents involving venomous animals had a rising temporal trend in the majority of Brazilian regions. Scorpion stings (51.2%), snake bites (17.4%) and spider bites (17.3%) accounted for 86% of reported accidents

**Implications for services**

In forthcoming years, health services will note an increase in accidents involving venomous animals, except for snake bites and caterpillar stings in the North, Northeast and South.

**Perspectives**

Guidance for production and distribution of antivenom sera in the country’s Federative Units, in addition to directing efforts towards given risk groups or locations.

## Introduction

Notification of accidents involving venomous animals is compulsory in Brazil, due to the magnitude of morbidity and mortality and their ability to produce temporary or permanent sequelae.^1^ Between 2001 and 2019 there were almost 140,000 accidents per year, comprising a public health problem that mainly affects the most vulnerable populations of the country, with snakebite, in particular, being classified by the World Health Organization as a neglected tropical disease.^1^ ^-^ ^3^

Brazil is of continental size, has tropical and subtropical zones, six types of biomes and several species of poisonous and venomous animals.^4^ As such, the epidemiological profile of accidents caused by venomous animals in Brazil varies both in space, between its geographic macro-regions, and also in time. These variations are related not only to type of vegetation, climate or relief, but also to actions of humans, climate change, disorderly urban growth and the elimination of natural predators, among other factors.^1^ ^,^ ^5^

Temporal trend analyses of accidents involving venomous animals are excellent strategies for analyzing how these phenomena oscillate in nature, allowing us to verify whether their incidence is falling, rising or stationary, in a given place.^6^

Although the literature contains several studies analyzing the epidemiological profile of venomous animal accidents in Brazil,^1^ ^,^ ^5^ ^,^ ^7^ at the time this report was completed, there were no up-to-date studies comparing temporal trends in the Brazilian geographic macro-regions.

Knowledge of the epidemiological profile is necessary both for guiding prevention policies for this type of accident and also for guiding the production and distribution of antivenom sera between the country’s Federative Units, in order to direct efforts towards given risk groups or locations.^7^

The objective of this study was to analyze the temporal trend of accidents involving venomous animals in Brazil from 2007 to 2019.

## Methods

This was an ecological study, taking the Brazilian geographic macro-regions as its units of analysis. Brazil has approximately 213 million inhabitants and covers an area measuring 8.5 million km^2^.^8^ As one of the largest and most populous countries in the world, Brazil has great biodiversity with regard to fauna and flora.^4^ The Brazilian Federation is comprised of 27 Federative Units, distributed between five geographic macro-regions: North (7), Northeast (9), Southeast (4), South (3) and Midwest (4). The national territory has five climate zones, defined as Equatorial, Temperate, Tropical - Central Brazil, Tropical - Eastern Northeast and Tropical - Equatorial Zone; and six classes of biomes, namely, Amazon, Atlantic Forest, *Cerrado*, *Caatinga*, *Pampas* and *Pantanal*.^4^

This study was performed on cases of accidents involving venomous animals in Brazil, recorded on the compulsory notification forms of the Notifiable Health Conditions Information System (*Sistema de Informação de Agravos de Notificação* - SINAN) between 2007 and 2019. The data on these cases were extracted from TABNET, a system available through the Brazilian National Health System Information Technology Department (*Departamento de Informática do Sistema Único de Saúde* - DATASUS), accessed on May 14, 2021.^3^

Using the TABNET platform, the data were filtered according to:

- year of notification (2007-2019);
- sex (male; female);
- age, stratified in age groups (in years: 0-4; 5-9; 10-14; 15-19; 20-39; 40-59; 60-69; 70-79; ≥ 80);
- region of residence (North; Northeast; Southeast; South; Midwest); and
- type of accident (caused by snakes, spiders, scorpions, caterpillars, bees and other creatures).

The "other" category refers to accidents caused by venomous animals that have less repercussion on people’s health, such as hymenopterans (ants and wasps), coleopterans (beetles), chilopodans (centipedes), diverse types of fish, cnidarians (jellyfish and Portuguese man-of-war); while "unknown" refers to accidents in which the animal that caused them is unknown. The latter category was not included in this study.

In order to calculate the incidence rate, demographic data were extracted from TABNET using filters for sex and age group.^9^ The incidence rates were obtained by dividing the number of new cases of each type of accident by the number of people at risk, according to the "sex" and "age group" variables. The ratio obtained was multiplied by 100,000 to arrive at the number of cases per 100,000 inhabitants.

Incidence rate association with sex was analyzed using relative risk (RR) calculations, taking male incidence as the numerator and female incidence as the denominator. The 95% confidence intervals (95%CI) for each RR were also calculated by Brazilian macro-region.

The case fatality ratio was estimated by dividing the number of deaths according to the type of accident reported (filtered from the TABNET system using the "case progression" variable) by the total number of reported accidents of the same type, multiplied by 100.

The Prais-Winsten linear regression method was used in the temporal trend analyses, taking the years evaluated, i.e. 2007 to 2019, as independent variables, when calculating annual percentage change (APC) and respective 95%CI. The venomous animal accident incidence rates were converted to the logarithmic scale (base-10).

Temporal trend was taken to be falling when the 95%CI values were negative; rising when the 95%CI values were positive; and stationary when the 95%CI included the value zero. A 5% significance level was adopted.^6^ The analyses were performed using STATA 13.

As this study was based on public domain data about people who are not identified in any way, it did not need to be submitted to a Research Ethics Committee for approval.

## Results

A total of 2,102,657 cases of accidents involving venomous animals were reported in Brazil between 2007 and 2019, with an annual average of 175,222. The most frequently occurring accident was scorpion sting (51.2%), followed by snakebite (17.4%) and spider bite (17.3%), accounting for 86% of cases. 

Some of these accidents were more frequent in certain regions of the country (Table 1). Scorpion stings were most frequent in the Northeast (68.6/100,000 inhab.), followed by the Southeast (41.2/100,000 inhab.) and the Midwest (26.5/100,000 inhab.), while snake bites predominated in the North (53.1/100,000 inhab.) and Midwest (19.0/100,000 inhab.) regions. The most predominant types of accident in the South were spider bites (61.3/100,000 inhab.), bee stings (9.2/100,000 inhab.) and caterpillar stings (6.3/100,000 inhab.). Accidents categorized as "other" were more frequent in the North (6.6/100,000 inhab.).

**Table 1:** Distribution of number of cases of accidents involving venomous animals (n), percentage of cases (%), annual mean (mean), number of deaths, case fatality ratio and incidence rate, by type of accident and national macro-region, Brazil, 2007-2019

Type of accident	Macro-regions and Brazil	n	%	Mean	Deaths	Case fatality ratio (%)	Incidence^a^
Scorpion sting	Midwest	53,153	2.5	4,089	69	0.12	26.5
	Northeast	496,039	23.6	38,157	492	0.09	68.6
	North	44,414	2.1	3,416	76	0.17	19.9
	Southeast	457,710	21.8	35,208	433	0.09	41.2
	South	26,247	1.2	2,019	7	0.02	6.9
	Brazil	1,077,563	51.2	82,889	1.077	0.09	32.6
Snakebite	Midwest	37,157	1.8	2,858	163	0.43	19.0
	Northeast	93,927	4.5	7,225	507	0.53	13.1
	North	117,128	5.6	9,010	495	0.42	53.1
	Southeast	84,115	4.0	6,470	232	0.27	7.6
	South	32,637	1.6	2,511	76	0.23	8.8
	Brazil	364,964	17.4	28,074	1.473	0.40	20.3
Spider bite	Midwest	8,142	0.4	626	18	0.22	4.0
	Northeast	17,812	0.8	1,370	50	0.28	2.4
	North	10,171	0.5	783	19	0.18	4.5
	Southeast	99,012	4.7	7,616	48	0.04	8.9
	South	228,325	10.9	17,564	48	0.02	61.3
	Brazil	363,462	17.3	27,959	183	0.05	16.3
Bee sting	Midwest	6,926	0.3	533	29	0.41	3.4
	Northeast	46,222	2.2	3,556	135	0.29	6.3
	North	7,230	0.3	556	26	0.35	3.1
	Southeast	59,969	2.9	4,613	150	0.25	5.4
	South	34,599	1.6	2,661	110	0.31	9.2
	Brazil	154,946	7.4	11,919	450	0.29	5.5
Other	Midwest	6,182	0.3	476	9	0.14	3.1
	Northeast	23,404	1.1	1,800	19	0.08	3.2
	North	14,903	0.7	1,146	11	0.07	6.6
	Southeast	29,095	1.4	2,238	19	0.06	2.6
	South	13,647	0.6	1,050	6	0.04	3.6
	Brazil	87,231	4.1	6,710	64	0.07	3.8
Caterpillar sting	Midwest	2,000	0.1	154	1	0.05	1.0
	Northeast	4,998	0.2	385	7	0.14	0.6
	North	2,886	0.1	222	-	0.00	1.2
	Southeast	20,996	1.0	1,615	7	0.03	1.9
	South	23,611	1.1	1,816	14	0.05	6.3
	Brazil	54,491	2.6	4,192	29	0.05	2.2

a) Incidence rate (cases/100,000 inhab.).

There was also variation in the case fatality ratio. It was higher for snakebite accidents (0.40%) and bee sting accidents (0.29%), in the Midwest and North regions, respectively (Table 1).

**Figure 1:**
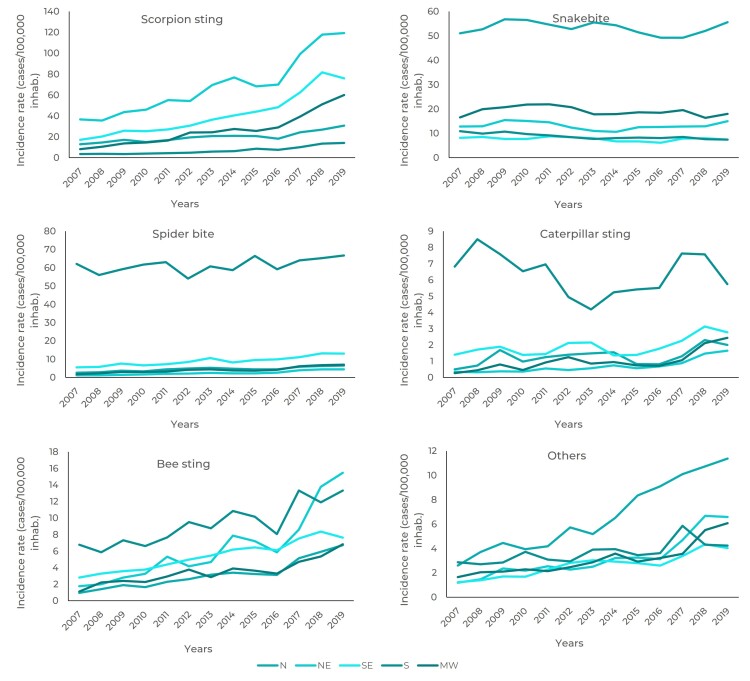
Time series of the incidence rate of accidents involving venomous animals (cases/100,000 inhab.), per national macro-region, Brazil, 2007-2019

Risk magnitude differed in space and time, as shown in Figure 1, in which it is evident, that in some regions the time series lines did not cross each other, e.g. scorpion stings in the Northeast, snake bites in the North, and spider bites and caterpillar stings in the South.

Of all the accident types observed, snake bites only had a falling temporal trend in the South; while in the remaining regions the trend was stationary. Caterpillar stings had a stationary trend in the North, Southeast and South. The remaining venomous animal accidents - including those classified as "other" - had a rising temporal trend (Table 2).

**Table 2:** Temporal trend of incidence of accidents involving venomous animals, by type of accident and national macro-region, Brazil, 2007-2019

Type of accident	Macro-region	APC^a^ (%)	95%CI^b^		Trend
			Lower limit	Upper limit	
Scorpion sting	Midwest	6.6	0.06	0.08	Rising
	Northeast	4.4	0.04	0.05	Rising
	North	2.6	0.18	0.34	Rising
	Southeast	5.4	0.05	0.06	Rising
	South	5.3	0.04	0.06	Rising
Snakebite	Midwest	-0.3	-0.01	0.01	Stationary
	Northeast	0.2	-0.01	0.02	Stationary
	North	0.0	-0.00	0.00	Stationary
	Southeast	-0.5	-0.01	0.00	Stationary
	South	-1.2	-0.02	-0.01	Falling
Spider bite	Midwest	4.4	0.03	0.06	Rising
	Northeast	5.0	0.04	0.06	Rising
	North	3.0	0.02	0.04	Rising
	Southeast	3.0	0.02	0.04	Rising
	South	0.4	0.00	0.01	Rising
Caterpillar sting	Midwest	5.9	0.03	0.09	Rising
	Northeast	5.5	0.04	0.07	Rising
	North	3.1	-0.00	0.06	Stationary
	Southeast	2.0	-0.00	0.04	Stationary
	South	-0.6	-0.03	0.02	Stationary
Bee sting	Midwest	4.6	0.03	0.06	Rising
	Northeast	7.2	0.06	0.09	Rising
	North	6.2	0.05	0.07	Rising
	Southeast	3.8	0.03	0.04	Rising
	South	2.7	0.02	0.03	Rising
Others	Midwest	4.2	0.03	0.05	Rising
	Northeast	5.5	0.04	0.07	Rising
	North	5.1	0.04	0.06	Rising
	Southeast	4.2	0.03	0.06	Rising
	South	2.0	0.01	0.03	Rising

a) APC: Annual percentage change; b) 95%CI: 95% confidence interval.

**Figure 2:**
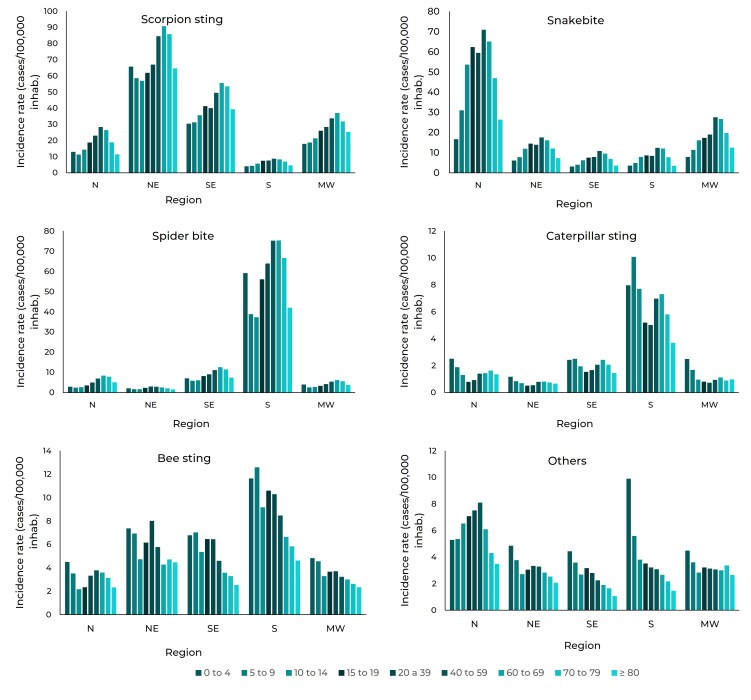
Incidence rate of accidents involving venomous animals (cases/100,000 inhab.), by age group and national macro-region, Brazil, 2007-2019

Scorpion stings, snake bites and spider bites occurred mostly among individuals of working age. The behavior of caterpillar stings was the opposite, with greater incidence among young and elderly people. On the other hand, risk of bee stings and "other" accidents was greater as age decreased, occurring more among young people and less among the elderly; the exception was the Northern region, where risk was higher among individuals of working age (Figure 2).

In the study period, we found higher incidence of all types of accidents with venomous animals among males, especially snake bites, the risk of which among males in Brazil as a whole was 3.47 times higher than among females (Table 3). In the Northeast, being male proved to be a protective factor against scorpion stings (RR = 0.8).

**Table 3:** Incidence of accidents involving venomous animals, by sex, relative risk and 95% confidence interval, according to type of accident and national macro-region, Brazil, 2007-2019

Type of accident	Macro-regions and Brazil	Incidence (cases/100,000 inhab.)			95%CI^b^	
		Male	Female	RR^a^	Minimum	Maximum
Scorpion sting	Midwest	28.5	26.1	1.09	1.07	1.10
	Northeast	61.3	76.8	0.80	0.79	0.80
	North	25.1	15.1	1.67	1.63	1.70
	Southeast	46.9	37.0	1.27	1.26	1.27
	South	7.7	6.4	1.21	1.18	1.23
	Brazil	41.9	41.0	1.02	1.02	1.03
Snakebite	Midwest	29.4	8.9	3.30	3.22	3.38
	Northeast	20.2	6.3	3.19	3.15	3.24
	North	83.4	22.4	3.72	3.67	3.77
	Southeast	12.0	3.5	3.41	3.35	3.46
	South	13.5	4.2	3.22	3.14	3.31
	Brazil	22.1	6.4	3.47	3.44	3.50
Spider bite	Midwest	4.7	3.6	1.30	1.24	1.36
	Northeast	2.6	2.4	1.06	1.03	1.09
	North	5.6	3.6	1.55	1.49	1.61
	Southeast	11.1	7.1	1.55	1.53	1.57
	South	60.8	62.0	0.98	0.97	0.99
	Brazil	14.9	13.1	1.14	1.13	1.15
Bee sting	Midwest	4.7	2.4	1.94	1.84	2.04
	Northeast	8.5	4.5	1.88	1.85	1.92
	North	4.2	2.3	1.81	1.73	1.90
	Southeast	7.2	3.8	1.90	1.87	1.94
	South	11.8	6.9	1.69	1.66	1.73
	Brazil	7.8	4.2	1.84	1.82	1.86
Caterpillar sting	Midwest	1.0	1.0	1.01	0.92	1.10
	Northeast	0.8	0.6	1.25	1.18	1.32
	North	1.4	1.2	1.12	1.04	1.21
	Southeast	2.2	1.6	1.38	1.34	1.42
	South	7.2	5.5	1.31	1.28	1.34
	Brazil	2.4	1.8	1.31	1.29	1.33
Others	Midwest	3.4	2.9	1.16	1.10	1.22
	Northeast	3.7	2.9	1.27	1.23	1.30
	North	9.2	4.3	2.15	2.08	2.22
	Southeast	3.1	2.2	1.39	1.35	1.42
	South	3.9	3.4	1.15	1.11	1.18
	Brazil	3.9	2.8	1.40	1.38	1.42

a) RR: Relative risk, taking the male sex as the reference group (male; female); b) 95%CI: 95% confidence interval.

## Discussion

Scorpion stings had the highest incidence among accidents caused by venomous animals in Brazil, in the period studied;^1^ and had a rising trend in the number of cases in all the country’s macro-regions. 

*Tityus serrulatus*, also known as the Yellow Scorpion, is the species most commonly associated with this type of accident^10^ and its spatial distribution has increased in all Brazilian regions, given its ease of adaptability to urban environments and rapid proliferation: *T. serrulatus* reproduces without needing to mate, adapts to different temperature zones and survives long periods without food or water.^11^ Attempts to control this scorpion species in Brazil have not been significantly successful.^11^

The Northeast region stood out as having the highest incidence of scorpion stings, and its victims were mostly female.^12^ This finding of our study is probably due to occupational and behavioral factors that can be found in the domestic environment, where females are more exposed.^13^

Scorpion stings occurred mostly among older individuals still of working age, demonstrating possible association of the home environment as a risk site for scorpion stings. Most of the victims of this type of accident were identified by another study as students, housewives, and retirees/pensioners.^14^

A study conducted in the state of Paraíba found no association between the socioeconomic factors analyzed and the geographic location of scorpion stinging; however, it is expected that higher rates of scorpion stinging will be found where housing conditions are poorer.^7^

The scorpion sting case fatality ratio in Brazil was 0.09% in the period studied, this being lower than that reported in other research, which found a 0.13% case fatality ratio in Brazil between 2009 and 2013.^12^ Although scorpion stings are frequent and generally only result in local clinical presentations, this is not the case regarding systemic manifestations, for which 5% of reported cases are severe and of these, 0.3% are fatal.^15^ The Northern region had a higher presented case fatality ratio for scorpion stings (0.17%), in relation to the other regions of the country. The fatal cases were found in the Amazon region, mainly following *Tityus metuendus* and *Tityus obscurus* stings.^16^

Regarding snake bites, the highest frequency of these accidents in the period studied was reported in the Northern region, thus corroborating the results of other studies.^17^ ^,^ ^18^ It is also noteworthy that the number of cases may be higher than found in this study, due to underreporting in the region, especially in relation to cases occurring in remote places.^18^

Regarding the profile of snakebite victims in Brazil, our study showed that most of the victims were male and of working age. This suggests occupational risk.^12^ ^,^ ^17^ Similar results were reported by another study, according to which males and rural workers are the most affected.^19^ Snakebite has also been described as an environmental risk accident for traditional population groups living in communities far from urban centers, such as nomads, indigenous people, hunters and gatherers.^20^ Still with regard to snake bites, they had a stationary temporal trend in almost all regions of the country; the only falling trend occurred in the Southern region.

Snake bites had the highest case fatality ratio among all accidents involving venomous animals in Brazil, with the Midwest and Northern regions standing out, this being where the *Pantanal* and Amazon biomes are located, respectively. These results were similar to those found by another study that analyzed venomous animal accidents in Brazil from 2007 to 2013.^12^ Several factors may be related to the snakebite case fatality ratio, such as the time elapsed between the accident and receiving health care, as well as difficulty in accessing a health service. In addition, other studies found that some victims or their legal guardians chose inadequate treatment measures, capable of making the case worse before getting to a health service, such as the use of homemade infusions, incision and/or application of substances at the site of the bite, tourniquet procedure, seeking help from a witch doctor, among others.^19^ ^,^ ^21^

Worldwide, snakebite affects about 2.7 million people, between 80,000 and 140,000 of whom die, mostly in India (50,000), followed by Pakistan (8,000) and Bangladesh (6,000). In the Americas, however, snakebite deaths are estimated in hundreds rather than thousands; in Brazil, there are 119 deaths per year.^2^ ^,^ ^12^ Some 87% of snakebite accidents in Brazil are related to vipers of the *Bothrops* genus.^22^

Spider bites had a rising temporal trend in the five Brazilian national macro-regions and accounted for 17.3% of notifications, with the Southern region standing out. Another study indicates that spider bite incidence in the South is ten times higher than in the other regions of Brazil:^1^ in Southern Brazil, the probability of spider bite does not appear to differ between sexes. This fact may possibly be related to its more frequent occurrence in urban areas and in households;^12^ spider bite in Southern Brazil mainly affects people of working age, while in the other regions of the country slightly higher relative risk is found for males. 

The spider bite case fatality ratio in Brazil as a whole was 0.05%, with the Northeast (0.28%) and Midwest (0.22%) regions standing out as accounting for the highest rates during the 13 years analyzed.^1^ ^,^ ^12^

The Northeast and South had the highest bee sting incidence rates; it should also be noted that all Brazilian regions showed a rising temporal trend. This problem has been recorded in the Brazil since 1956, after the accidental release of Africanized bees that spread throughout the Americas and became a public health problem in the countries where they formed colonies.^23^ Africanized honey bees are significantly more defensive than other bees, attack with little provocation, attack in greater numbers, pursue their victim for longer periods, and apparently release greater amounts of venom.^23^

Individuals aged 0 to 9 years old and males were the most affected by bee stinging. In all regions of the country, the data suggest no causality between this type of accident and the economically active population. A study carried out in a city in Northeast Brazil indicated that most bee sting cases occurred in urban areas and were unrelated to work.^24^

The bee sting case fatality ratio in Brazil as a whole was 0.29%, with higher ratios being found in the Midwest (0.41%) and Northern (0.35%) regions in the period studied. A study covering the period from 2001 to 2012 found case fatality ratios ranging from 0.3% to 0.4% in all regions, pointing to the severity of outcomes associated with the large amount of venom injected as a result of multiple stings. This can result in anaphylactic reaction.^1^ Another study found that bee stings increased by more than 200% in the ten years between 2009 and 2019, with case fatality being higher in older people and people living in the Northeast region.^25^

Bee stings are expected to be less fatal than snake bites or scorpion stings. However, the bee sting case fatality ratios found in this study indicate similar or even higher values than the snakebite and scorpion sting case fatality ratios in several Brazilian regions. One possible hypothesis for this evidence is the higher frequency of notification of the most severe bee stings (involving multiple stings and allergic individuals), as well as underreporting of mild cases in which the victim does not seek medical care, thus directly affecting the case fatality ratio calculation.

Caterpillar stings showed a stationary temporal trend in the North, Southeast and Southern regions, and a rising trend in the Midwest and Northeast regions. According to other studies, in the future, caterpillar stings will have greater incidence and broader geographic distribution, given (i) global warming, (ii) destruction of their natural habitat, forcing caterpillars to live in other areas, especially in trees located in urban surroundings, and (iii) the killing of natural predators due to extensive pesticide use.^26^

Caterpillar stings had the lowest incidence in relation to the other accidents involving venomous animals analyzed in this study. However, although underreported, this is perhaps the most common accident involving venomous animals, especially in tropical climates, not only through direct contact but also through indirect contact (airborne), and can result in diversified clinical pictures.^26^

The Southern region accounted for the highest caterpillar sting incidence rates. This result has also been found in other studies. It is possible that changes caused by humans to land use have led to the decrease of tree hosts and have consequently led to the need for their adaptation and migration to new host environments, such as orchards close to urban areas.^27^

There was little difference in caterpillar sting incidence between the sexes. In effect, incidence was higher among children, regardless of the region of the country, and a possible explanation for this result could be the fact that the younger age groups are more exposed, driven by curiosity to catch or touch caterpillars. Among adults, it is possible that these accidents are related to occupations that result in contact or proximity to plants and take place outdoors.^28^

Accidents recorded as "other" on the SINAN system, caused by hymenopterans, coleopterans, chilopodans, cnidarians, diverse types of fish and other creatures, had a rising temporal trend in all the regions of the country, but accounted for less than 5% of reported cases. In the North, their highest incidence related to male, suggesting occupational accidents; in the other regions, there was little distinction between the sexes; children under 5 years of age were the most affected, probably because they are more defenseless in relation to ants, wasps, beetles and centipedes, among others. It is assumed that cases classified as "other" occur in the home environment. Incidentally, the scarcity of studies dedicated to interpreting this type of data is noteworthy.

Based on the results of this study’s temporal analysis, an annual increase in all accidents caused by venomous animals, to be cared for in health services, is projected for the coming years. The exceptions to this forecast are snakebite; and caterpillar stinging in the North, Northeast and Southern regions of the country. 

In order to mitigate this type of health problem, whether in urban or rural environments,taking into account that many accidents result from occupational risk and are therefore preventable, it is recommended that special attention be paid to the use of personal protective equipment (PPE) in certain work activities. In order to reduce household accidents, fumigation of domestic environments, constant cleaning of households and elimination of possible venomous animal hiding places are recommended.^17^

Research with smaller-scale geographic units, such as states and municipalities, is also recommended, since it may demonstrate patterns different from those found at the macro-regional level. 

We conclude that there was a rising temporal trend for most accidents with venomous animals during the study period. The spatial distribution of these accidents occurred heterogeneously and showed distinct epidemiological patterns
